# Latency profiles of full length HIV-1 molecular clone variants with a subtype specific promoter

**DOI:** 10.1186/1742-4690-8-73

**Published:** 2011-09-16

**Authors:** Renée M van der Sluis, Georgios Pollakis, Marja L van Gerven, Ben Berkhout, Rienk E Jeeninga

**Affiliations:** 1 Laboratory of Experimental Virology, Department of Medical Microbiology, Centre for Infection and Immunity Amsterdam (CINIMA), Academic Medical Centre, University of Amsterdam, Meibergdreef 15, 1105 AZ Amsterdam, the Netherlands

## Abstract

**Background:**

HIV-1 transcription initiation depends on cellular transcription factors that bind to promoter sequences in the Long Terminal Repeat (LTR). Each HIV-1 subtype has a specific LTR promoter configuration and even minor sequence changes in the transcription factor binding sites (TFBS) or their arrangement can impact transcriptional activity. Most latency studies have focused on HIV-1 subtype B strains, and the degree to which LTR promoter variation contributes to differences in proviral latency is therefore largely unknown. Latency differences may influence establishment and size of viral reservoirs as well as the possibility to clear the virus by therapeutic intervention.

**Results:**

We investigated the proviral transcriptional latency properties of different HIV-1 subtypes as their LTRs have unique assemblies of transcription factor binding sites. We constructed recombinant viral genomes with the subtype-specific promoters inserted in the common backbone of the subtype B LAI isolate. The recombinant viruses are isogenic, except for the core promoter region that encodes all major TFBS, including NFκB and Sp1 sites. We developed and optimized an assay to investigate HIV-1 proviral latency in T cell lines. Our data show that the majority of HIV-1 infected T cells only start viral gene expression after TNFα activation.

**Conclusions:**

There were no gross differences among the subtypes, both in the initial latency level and the activation response, except for subtype AE that combines an increased level of basal transcription with a reduced TNFα response. This subtype AE property is related to the presence of a GABP instead of NFκB binding site in the LTR.

## Background

Combined antiretroviral therapy (cART) is able to suppress the HIV-1 plasma RNA load in patients to undetectable levels. Unfortunately, the treatment does not lead to a complete eradication of the virus from the infected individual. Even after many years of successful cART, the virus rebounds from latently integrated proviral DNA reservoirs and re-establishes systemic infection upon interruption of therapy [1-4]. HIV-1 proviral latency may be an effective means to evade the immune system, since the infected cell will go unnoticed by the immune system as long as viral antigens are not expressed and presented. The pool of latent proviruses is established early during infection and forms a steady source of proviral DNA that can last a lifetime for infected individuals [5-7]. The majority of the latent proviruses reside in long-lived memory CD4^+ ^T cells, but other cellular reservoirs, such as monocytes, macrophages and dendritic cells, can also harbor latent proviruses [8-11]. HIV-1 latency remains a formidable barrier towards virus eradication as therapeutic attempts to purge these reservoirs have been unsuccessful [3,9,12,13].

Previously reported contributors to proviral latency include suppressive effects of cellular microRNAs, an impaired viral Tat-TAR axis, and epigenetic silencing via histone modification and DNA hypermethylation [14-18]. Most of these modulators have been studied in artificial cell line models for HIV-1 latency, but some of these mechanisms were found to be operational in resting CD4^+ ^T-cells from HIV infected patients [19,20]. HIV-1 transcriptional activation from latency depends on cellular transcription factors that bind to the Long Terminal Repeat (LTR) promoter. Differences in promoter activity among the HIV-1 subtypes have been reported, consistent with the fact that their LTRs have specific configurations of transcription factor-binding sites (TFBS), including variation in the number and sequence of NFκB, STAT5 and C/EBP sites [21-25]. Such subtype-specific promoter characteristics correlate with significant differences in terms of viral replication kinetics and the response to environmental changes [26]. The interaction between cell type specific transcription factors and LTR sites is crucial for the regulation of virus expression and possibly proviral latency. Therefore, we investigated the influence of the subtype-specific promoters on HIV-1 transcriptional latency in a single round infection-based latency assay model.

We demonstrate that the majority of the HIV-1 infected T cells initiate viral production only after TNFα activation. There were no gross differences in latency and activation properties among the subtypes, except for subtype AE. This subtype combines increased levels of productive infection with a reduced TNFα response, which correlates nicely with the presence of a GABP instead of an NFκB transcription factor binding site in its LTR.

## Results

### Latency model

**Figure 1:**
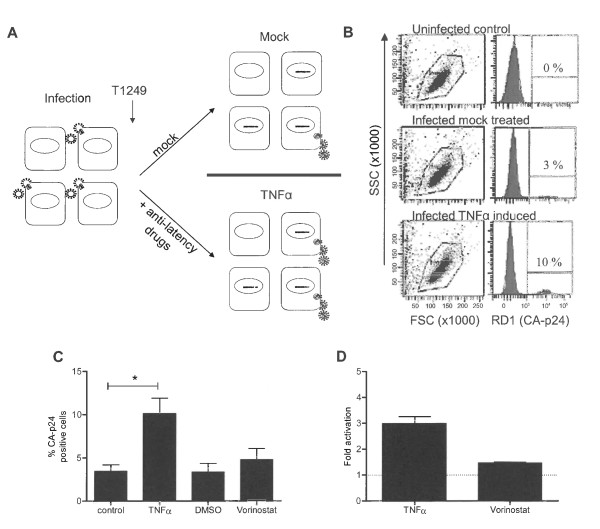
**HIV-1 latency assay**. **A: **Schematic of the HIV-1 latency assay. SupT1 T cells are infected with HIV-1 for 4 hours, free virus is washed away, and the fusion inhibitor T1249 is added to prevent new infections. Infected cultures are split 24 hours after infection into a mock and anti-latency drug treated culture. Cells are harvested 24 hours after treatment, stained for intracellular CA-p24 and analyzed by FACS. The fold activation (as viral latency marker) is the ratio of CA-p24 positive cells in the drug versus mock treated sample. **B: **Representative FACS analysis. Live cells are gated using the Forward/Sideward scatter (FSC/SSC) and scored for CA-p24 positivity in the RD1 channel. **C: **Latency assay: percentages of CA-p24 positive cells in control (mock treated), TNFα treated, Vorinostat treated and DMSO treated (mock for Vorinostat treated) cultures. The results presented are the average values of two independently produced virus stocks, which were both used in two independent infections. Significant difference (*) was determined with the student T-test (Graphpad Prism). **D: **The fold activation (percentage CA-p24 positive cells in drug induced culture versus mock culture).

We have previously described a single round infection assay to determine HIV-1 transcriptional latency, which occurs even in actively dividing T cells [27]. In this assay the SupT1 T cell line is infected with HIV-1_LAI _for 4 hours after which the fusion inhibitor T1249 is added to prevent new infections (Figure 1A). The culture is split 24 hours post infection and either treated with anti-latency drugs or not (mock). Treated cells are harvested 24 hours later, fixed, stained for intracellular CA-p24 and analyzed by FACS. The living cell population was subsequently scored for intracellular CA-p24 production (Figure 1B).

First, we optimized the latency assay to score the impact of cellular stimuli on the HIV-1 subtype B strain LAI and tested the cytokine TNFα as anti-latency drug. The subtype B LTR promoter contains two NFκB binding sites through which transcription can be triggered by activation of the NFκB pathway with TNFα [28-32]. In addition, NFκB stimulates transcriptional elongation by RNA Polymerase II through binding of the pTEFb cofactor [33]. We also tested Vorinostat (SAHA), an inhibitor of histone deacetylases, which creates a more open nucleosome conformation thereby making the HIV-1 promoter more accessible to transcription factors [34,35]. In the mock treated culture, 3.4% of the cells produced CA-p24, which increased to 10.1% in the TNFα treated culture (Figure 1C). The ratio between TNFα and mock treated cultures ("fold activation") is used as a measure of viral latency. TNFα treatment induced a significant 3-fold increase in the percentage of CA-p24 positive cells (Figure 1D). In this assay, we only scored the productively infected cells, either directly or after drug treatment. We did not detect unresponsive or defective proviral genomes. The results indicated that there are at least 3 times as many latent integration events compared to productive integrations of an intact provirus in SupT1 T cells that can be activated upon TNFα treatment. Vorinostat has a less pronounced effect as CA-p24 positivity is increased from 3.4% to 4.8%, yielding a 1.5-fold activation. Combinations of both anti-latency drugs did not yield any further significant increases in activation over the TNFα effect (results not shown). In this setting of recently integrated proviruses, Vorinostat has no additional effect over the already strong effect of TNFα. These results do not necessarily mean that all latently integrated proviruses are activated. It is likely that we cannot activate all latently integrated proviruses. Even latency studies using (clonal) cell lines, with each individual cell containing a latently integrated provirus, cannot purge 100% of the proviruses out of latency using a mixture of anti-latency drugs [18,27,28,36-40].

TNFα stimulation affects the process of HIV-1 transcription, but might also affect the amount of proviruses generated upon cell stimulation. To exclude an effect of TNFα induction on the efficiency of reverse transcription and provirus formation, we performed a real-time TaqMan assay to score the average number of HIV-1 DNA copies per cell. We measured no difference between TNFα induced and mock treated after 24 hours of stimulation (data not shown), demonstrating that TNFα does not influence the efficiency of reverse transcription and/or the amount of viral DNA that is produced, consistent with an exclusive impact on LTR-mediated transcription.

### Linear range of the latency model

**Figure 2:**
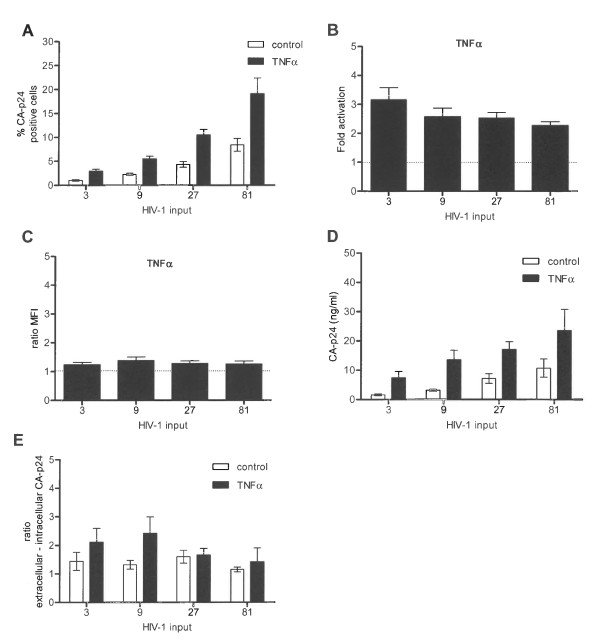
**Performance of the HIV-1 latency assay**. **A: **Average percentages of CA-p24 positive cells as determined by FACS in SupT1 T cells infected with increasing concentrations of HIV-1_LAI _(ng/infection). Cells were either mock treated or TNFα induced. **B: **Fold activation from latency with increasing viral input. **C: **Ratio MFI of TNFα induced versus mock cultures. **D: **Extracellular CA-p24 concentrations in TNFα induced and mock treated cultures. **E: **The concentration of extracellular CA-p24 was corrected for the percentage of intracellular CA-p24 positive cells. Results are shown as the ratio of extracellular versus intracellular CA-p24. The results presented are the average values that were obtained with three independently produced virus stocks, and each stock was used for two independent infections.

To investigate the linear range of this latency assay, we infected SupT1 cells with increasing amounts of subtype B and determined the percentage of CA-p24 positive cells with and without TNFα activation. Upon increasing the virus input, more cells become infected and TNFα activation yielded an increase in the percentage of CA-p24 positive cells (Figure 2A). The fold activation, however, gradually decreased with increasing viral input (Figure 2B). A possible explanation for this is that at high viral input cells become infected by multiple viruses, with transcriptionally active proviruses 'overruling' silent copies. Such cells will be quantified as CA-p24 positive, leading to an underestimation of latent proviruses. At the other end of the spectrum, results became more variable and thus less reliable when less than 1% CA-p24 positive cells were scored in the non-treated control. In subsequent infection experiments, we have titrated the virus such that 1 to 5% of the cells became CA-p24 positive without activation.

The results presented thus far demonstrate that TNFα treatment increases the number of CA-p24 producing cells. To determine whether cells also start producing more CA-p24 upon TNFα stimulation, we analyzed the mean fluorescent intensity (MFI) of the CA-p24 positive cells. As with fold activation, we used MFI ratios of induced to non-treated cultures to determine the relative change in intracellular CA-p24 production level. This MFI ratio upon TNFα treatment was close to 1, indicating that TNFα treatment does not increase the viral gene expression levels, but only the number of active proviruses (Figure 2C). To check whether perhaps more CA-p24 was secreted, the concentration of CA-p24 in the culture supernatant was quantified by ELISA. The TNFα induced cultures showed increased CA-p24 levels in the supernatant since TNFα induced more cells to produce CA-p24 (Figure 2D). When we correlated the extracellular CA-p24 levels with the number of CA-p24 producing cells, an increase was observed upon TNFα induction in the cultures infected with 3 ng and 9 ng CA-p24 as viral input for infection. However, these differences were not statistically significant (Figure 2E). Thus, the latency model optimized for the wild-type HIV-1 subtype B allows one to score for activation of latent proviruses.

### Latency over time

**Figure 3:**
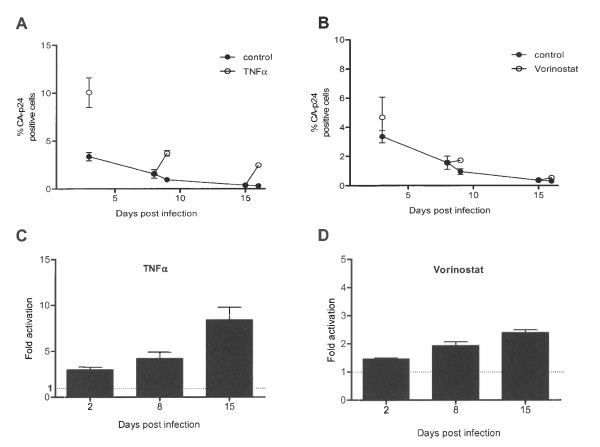
**HIV-1 latency over time**. **AB: **SupT1 T cells were infected with HIV-1_LAI_. On day 2, 7 and 14 the culture was split and either mock treated, induced with anti-latency drugs (TNFα or Vorinostat) or passaged and cultured for another week, when the protocol was repeated. Cells were harvested 24 hours after treatment (day 3, 8 and 15 respectively). Percentages of CA-p24 positive cells were determined by FACS. **CD: **The fold activation from latency. The results presented reflect the average of two independently produced virus stocks, and each was used in two independent infections.

We were interested in monitoring proviral latency over an extended time window. The fusion inhibitor T1249 remained present in these cultures to prevent spreading of the input virus. A sample of the cultures was split on day 2, 7 and 14 and either TNFα or mock treated. The cells were harvested 24 hours later and analyzed by FACS. The percentage of CA-p24 positive cells in the mock culture decreased gradually over time from 3.3% to 0.4% (Figure 3A). The TNFα-treated level of CA-p24 positive cells also decreased, but less dramatically. This indicates that the fold activation as latency measurement increased considerably from 3-fold on day 3 to 10-fold on day 15 (Figure 3C). However, as described above, a too low percentage of CA-p24 positive cells yields less reproducible values, and we therefore decided to focus on the latency measurement after 24 hours. Nevertheless, the data in Figure 3C do clearly demonstrate that latency gets more dramatic over time.

Similar experiments were performed with the HDAC inhibitor Vorinostat (Figure 3B and 3D). Over time, both mock and Vorinostat treated cultures showed a decrease in number of CA-p24 positive cells, and the activation from latency increased from 1.5-fold on day 3 to 2.4-fold on day 15.

### Latency properties of different HIV-1 subtypes and T cell lines

**Figure 4:**
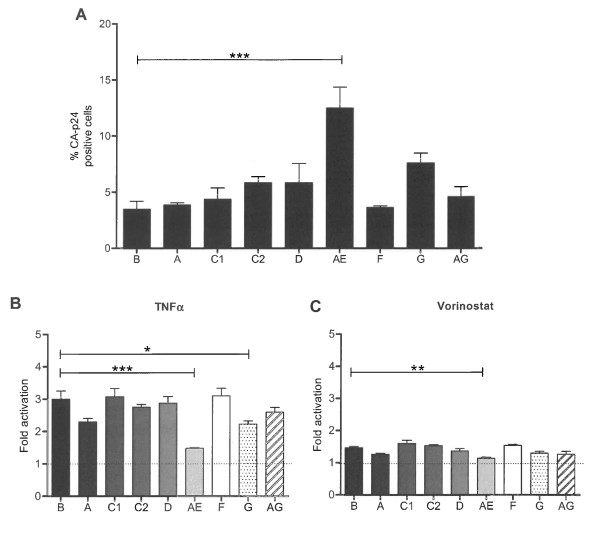
**Influence of the HIV-1 promoter on proviral latency**. **A: **Viruses containing the indicated subtype specific LTR promoter were used in the latency assay. **BC: **Fold activation from latency with TNFα (B) and Vorinostat (C). The results are the average values that were obtained with two independently produced virus stocks, each tested in two independent infections. P values * = p < 0.05, ** = p < 0.01, *** = p < 0.001 were determined with the One Way ANOVA (Graphpad Prism).

To investigate the influence of the subtype-specific promoter on proviral latency, SupT1 cells were infected with an equal amount of the different viruses. Without inducers, subtype B yielded 3.4% CA-p24 positive cells, which represented the basal transcription level (Figure 4A). The subtypes A, C, D, F and AG yielded very similar percentages, but subtypes G and AE demonstrated an increase in their basal transcription activity. Upon TNFα activation, percentages of CA-p24-producing cells increased for all subtypes, with an activation of around 3-fold, except for subtypes G and AE (Figure 4B). Activation of subtype G was only 2.2-fold, and subtype AE was even less potent at 1.5-fold. Thus, subtypes with a higher basal transcription level were less inducible with TNFα. In other words, subtypes AE and G proviruses were less prone to become latent. The HDAC inhibitor Vorinostat induced activation from latency for all subtypes, but with a reduced potency compared to TNFα (Figure 4C). However, the same subtype trends were apparent, with the highest activation for subtype C and the lowest induction for subtype AE.

We already showed that subtype B exhibits a more severe latency profile over time. The subtype-specific cultures were also assayed over longer periods, and the latent provirus was activated with TNFα. Subtype G, which exhibits reduced latency compared to B, also obtains a more dramatic latency profile over time (Additional File 1 Fig. S1A). However, subtype AE activation from latency remains close to 1.5-fold, the same latency value as measured at day 2 post infection. Thus, subtype AE infection starts with higher basal transcription levels, exhibits a reduced latency and the AE latency profile does not become more dramatic over time as observed for the other subtypes.

Similar experiments were performed with the HDAC inhibitor Vorinostat. As expected, activation does not reach similar levels as TNFα treatment (Additional file 1, Fig. S1B). Again, subtype AE was less prone to activation by Vorinostat as compared to the other subtypes.

**Figure 5:**
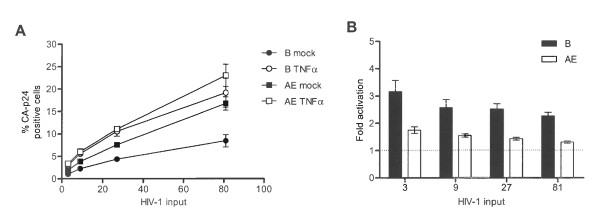
**Latency profile comparison of HIV-1 subtypes AE and B**. **A: **SupT1 T cells were infected with increasing virus concentrations of subtype B or AE (3, 9, 27 and 81 ng/infection CA-p24) in the presence or absence of TNFα. **B: **Fold activation with TNFα for subtypes AE and B with the indicated viral inputs. The results presented reflect the average of three independently produced virus stocks and each stock was used in two independent infections. The HIV-1 input (ng/infection) was based on CA-p24 ELISA.

Compared to subtype B, AE has increased basal transcription levels and shows reduced latency. To ensure that the measurements were still in the linear range of the assay, SupT1 cells were infected with different amounts of subtype AE or B virus. As expected, the basal percentage of CA-p24 positive cells was always higher for AE than B (Figure 5A). Likewise, the fold activation was always higher for B than AE. Additionally, the TNFα induced activation from latency for subtype AE remained around 1.5-fold (Figure 5B). These results demonstrate that subtype AE measurements are within the linear range of the assay and, more importantly, that AE is less responsive to TNFα induction since the AE promoter activity is higher compared to B at basal settings.

To investigate if the obtained results are specific for SupT1 cells, we repeated the experiments in Jurkat cells because many HIV-1 latency studies have been performed using this T cell line [17,18,30,38]. The percentage of CA-p24 positive cells without induction, reflecting the basal transcription level, was slightly higher for AE as compared to B (1.2 and 1.0% respectively, Additional File 2, Fig. S2A). However, activation from latency by TNFα induction was significantly higher for B than for AE (Additional File 2, Fig. S2B). These results demonstrate that subtype AE also exhibits reduced latency compared to subtype B in the Jurkat T cell line.

### NFκB versus GABP

**Figure 6:**
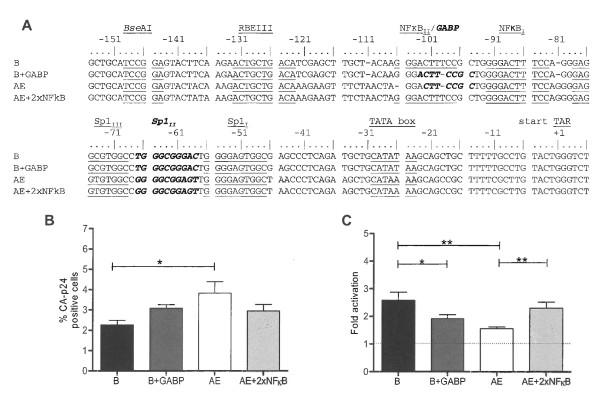
**LTR promoter elements of subtypes B and AE**. **A: **The core promoter elements in the LTR of subtypes B and AE. Indicated transcription factor binding sites are: RBEIII, NFκB, GABP, Sp1 and the TATA box. *Bse*A1 indicates the recognition site for the endonuclease used for molecular cloning. **B: **Percentages of CA-p24 positive cells without induction determined by FACS. C: Fold activation by TNFα induction. The results are presented as the average values of three independently produced virus stocks, each tested in two independent infections. P values: * = p < 0.05, ** = p < 0.01, *** = p < 0.001.

Infection of T cells with HIV-1 subtype AE yields more CA-p24 producing cells than equivalent infections with subtype B. On the other hand, TNFα induced activation from latency is reduced for AE compared to B, which thus yield similar end production levels. Arguably, the AE LTR might be less prone to become silenced due to the presence of the unique GABP binding site instead of the regular second NFκB site present in the other subtypes. The GA binding protein (GABP) complex is composed of two subunits. GABPα binds to the DNA and GABPβ contains the transcriptional transactivation domain. This transcription factor has been demonstrated to have a role in basic cellular functions and has recently been described to have a critical role in differentiation and maintenance of hematopoietic progenitor cells [41]. To investigate if the GABP binding site is responsible for the increased basal transcription level and decreased TNFα response, we made several alterations in the two promoters and tested latency properties (Figure 6A). Replacing the GABP with a second NFκB binding site in the AE promoter (AE+2xNFκB) slightly decreased the basal transcription level and subsequently increased the TNFα response (Figure 6BC). Activation from latency increases significantly from 1.6-fold for AE to 2.3-fold for AE+2xNFκB. To examine if GABP is the sole factor that is responsible for this effect, we subsequently converted the upstream NFκB into a GABP site in subtype B (B+GABP). The basal transcription level increased from 2.3 for B to 3.1 for B+GABP, which is not statistically significant. However, the GABP insertion altered the TNFα response which decreased significantly from 2.6-fold for B to 1.9-fold for B+GABP. Taken together, these results demonstrate that the NFkB to GABP conversion partially explains the higher basal activity combined with lower response to activation but that GABP is probably not the sole responsible factor.

## Discussion

HIV-1 proviral latency is a major barrier towards virus eradication from the infected patient. This latent virus reservoir is established early in infection [7,12]. In this manuscript, we introduce a latency model system that creates the opportunity to study proviral latency in actively dividing T cells. The model is based upon a single round infection in combination with FACS analysis to determine virus production per cell. A major advantage of this model system is the use of wild type HIV-1 instead of plasmids or sub-genomic reporter constructs. Additionally, the infected cells do not need to be cultured for an extended period, thus allowing one to study latency directly after infection in wild type cells without selection, in contrast to previous described latency model cell lines such as U1, ACH-2, OM-10.1 and J-Lat [42-45]. In principle, our method can be applied to any type of cell susceptible to HIV-1 infection.

In an acute HIV-1 infection model with the SupT1 T cell line, we demonstrate that a low percentage of the infected cells is able to express the integrated provirus. The majority of infected cells carry a latent provirus, which we could identify upon provirus activation from latency by TNFα. For HIV-1 subtype B, we measured a 3-fold increase in the percentage of CA-p24 positive cells. However, the amount of viral CA-p24 production per producing cell did not increase. The HDAC inhibitor Vorinostat was also able to activate latent provirus, although less efficient than TNFα. Combinations of both anti latency drugs did not yield any further significant increases in activation.

Culturing the infected cells over an extended period caused a relative decrease in the number of CA-p24 positive cells. Transcriptional silencing of active proviruses seems unlikely because we use actively dividing T cells. It seems more likely that the decrease in percentage of CA-p24 positive cells is due to cell death induced by HIV-1 [46]. In addition, as HIV-1 induces cell cycle arrest [47], virus producing cells can no longer proliferate, and thus their percentage will gradually decline relative to uninfected cells. Considering both factors, the decrease in CA-p24 positive cells seems relatively slow. This might be due to replenishment of the CA-p24 producing population by cells with a latent provirus that becomes transcriptionally active, which is in agreement with the stochastic model of HIV-1 reactivation [48-50]. We demonstrate that latent proviruses remain present, as TNFα was still able to induce a significant increase in the CA-p24 positive population at day 15. In fact, activation increased from 3-fold at day 2 to 10-fold at day 15. However, the absolute percentage of CA-p24 positive cells obtained upon TNFα treatment decreases over time. The latter observation further supports the hypothesis of stochastic activation of latently integrated provirus, causing this population to slowly decline. Alternatively, some of the latent proviruses may become silenced more stringently over time such that TNFα no longer suffices for activation. We are currently studying both options.

We have analyzed the promoter of different HIV-1 subtypes and observed that subtypes A, C, D, F and AG have similar latency profiles as B. Interestingly, the promoter of subtype C contains a third consensus DNA sequence for NFκB binding [51]. Although it has not been shown that this site is actually bound by NFκB, experiments with LTR-luciferase reporter plasmids have demonstrated that subtype C promoter activity is increased compared to subtype B upon stimulation with TNFα or other NFκB activators [22,25,38,52-54]. Viral fitness studies have also demonstrated relative advantages for the subtype C promoter in a TNFα-rich environment [26]. However, in terms of proviral latency, we did not observe a significant difference between C and the other subtypes.

Subtype AE clearly exhibits a reduced level of latency, which correlates with a GABP instead of NFκB transcription factor binding site in the LTR. The GABP-to-NFκB mutation in the AE promoter only slightly reduced the basal transcription level but did restore the TNFα response. The reciprocal experiment, the NFκB-to-GABP switch in subtype B, did not alter the basal levels but did significantly reduce the TNFα response. Thus, the GABP site is an important (but probably not the sole) determinant of the subtype AE specific properties. We are currently investigating other sequence variations between subtype AE and B to further elucidate the observed differences.

Opijnen *et al*. demonstrated that the LTR impact on viral replication depends on the cellular environment, either by host cell type or the presence of activators [26]. Subtype AE out-competed all other subtypes in the SupT1 T cell line. However, subtype AE became the worst competitor upon TNFα addition. Our observations indicate that the AE promoter has an advantage over the other subtype-specific promoters in a TNFα-poor environment, in part due to the unique GABP site causing AE to become latent less frequently than the other subtypes. Interestingly, a long-term culture of SupT1 T cells infected with a Tat-defective poorly replicating, HIV-1_LAI _variant, resulted in a spontaneous NFκB-to-GABP conversion, which significantly increased viral replication [55]. This also indicates strong differences between subtypes AE and B in their replication and latency profiles. Because subtype AE proviruses are less prone to become latent, this may translate in higher chances of purging the reservoir. In other words, a cure may be within closer reach for subtype AE infected individuals.

## Conclusions

We used a novel model of HIV-1 infection to study proviral latency in actively dividing T cells, of which the majority only support viral gene expression after TNFα activation. We measured no gross differences among the HIV-1 subtypes, both in the initial latency property and the activation response, except for subtype AE that combines an increased level of basal transcription with a reduced TNFα response. This subtype AE property is related to the presence of a GABP instead of NFκB binding site in the viral LTR promoter.

## Methods

### Cells and viruses

HEK 293T cells were grown as a monolayer in Dulbecco's minimal essential medium supplemented with 10% (v/v) fetal calf serum (FCS), 40 U/ml penicillin, 40 μg/ml streptomycin, 20 mM glucose and minimal essential medium nonessential amino acids at 37°C and 5% CO_2_. The human T lymphocytic cell lines SupT1 (ATCC CRL-1942) [56] and Jurkat (ATCC TIB-152) were cultured in advanced RPMI 1640 medium (Gibco BRL, Gaithersburg, MD) supplemented with 1% (v/v) FCS, 40 U/ml penicillin, and 40 μg/ml streptomycin at 37°C and 5% CO_2_. HIV-1 infections were performed with 293T produced virus stocks of the different HIV-1 molecular clones. The cells were transfected with plasmid DNA of the HIV-1 LAI molecular clone [57] or derivates thereof by the calcium phosphate method as described previously [58]. LTRs from patient isolates representing subtype A, C, D, AE (CRF_01), F, G and AG (CRF_02) were selected as being representative of the viral quasi species in the patient and the HIV-1 subtypes [22]. These subtype-specific LTRs were cloned into the common viral backbone of HIV-1_LAI _(subtype B). The recombinant viruses are isogenic except for the core promoter region containing the major TFBS, thus preventing differences in fusion, integration etc. The variable LTR region spans only 150 bp, containing the major TFBS, but still encoding a subtype B TAR hairpin. The concentration of the produced virus stocks was determined by CA-p24 ELISA.

### Reagents

TNFα (Invitrogen PHC3015) was prepared in sterile milliQ H_2_O (stock solution 10 μg/ml) and used at a final concentration of 50 ng/ml. Fusion inhibitor T1249 (WQEWEQKITALLEQAQIQQEKNEYELQKLDKWASLWEWF, Pepscan Therapeutics BV, Lelystad, the Netherlands) was obtained as a 10.000 × stock solution of 1 mg/ml. Vorinostat was donated by Frank Dekker (Groningen University, the Netherlands). The lyophilized powder was dissolved in DMSO (2 mM stock solution) and used at a final concentration of 0.3 μM.

### HIV-1 latency assay

#### Single round infection assay

SupT1 or Jurkat T cells (0.5 × 10^6 ^cells) were infected with virus stocks of the primary CXCR4-using LAI isolate or derivatives containing a subtype-specific 3'LTR. Excess virus was washed away after four hours and the cells were cultured in the presence of the fusion inhibitor T1249 to block all subsequent viral entry. The cultures were split 24 hours post-infection, and TNFα was added to a single culture. After another 24 hours, we measured intracellular CA-p24 by FACS analysis and extracellular CA-p24 production in the culture medium by ELISA. To equalize infections, input CA-p24 was kept similar among subtype-specific infections and conditional medium was added to reach a 200 μl infection volume.

#### Intracellular CA-p24 staining and fluorescence-activated cell sorting

Flow cytometry was performed with RD1- or FITC-conjugated mouse monoclonal anti-CA-p24 (clone KC57, Coulter). Cells were fixed in 4% formaldehyde for at least 5 min at room temperature, washed with FACS buffer (PBS with 10% FCS) and kept at 4°C. The cells were washed with BD Perm/Wash™ buffer (BD Pharmingen) and stained for at least 30 minutes at 4°C with the appropriate antibody diluted 1:100 in BD Perm/Wash™ buffer. Excess antibody was removed by washing the cells with BD Perm/Wash™ buffer and the cells were resuspended in FACS buffer. Cells were analyzed on a BD FACSCanto II flow cytometer with BD FACSDiva Software v6.1.2 (BD biosciences, San Jose, CA). Cell populations were defined based on forward/sideward scattering. Results from different assays were corrected for between-session variation with the factor correction program [59].

#### Extracellular CA-p24 ELISA

Culture supernatant was heat inactivated at 56°C for 30 minutes in the presence of 0.05% Empigen-BB (Calbiochem, La Jolla, USA). The CA-p24 concentration was determined by a twin-site ELISA with D7320 (Biochrom, Berlin, Germany) as capture antibody and alkaline phosphatase-conjugated anti-p24 monoclonal antibody (EH12-AP) as detection antibody. Quantification was performed with the lumiphos plus system (Lumigen, Michigan, USA) in a LUMIstar Galaxy (BMG labtechnologies, Offenburg, Germany) luminescence reader. Recombinant CA-p24 produced in a baculovirus system was used as a standard.

### Plasmids

Cloning of the different subtype specific LTRs (A, C1. C2, D, AE = CRF01, F, G and AG = CRF02) into the full length LAI molecular clone has been described previously [22]. Subtype C1 and C2 do not refer to the different C subclusters, C and C', but resemble two variants within subcluster C [60,61]. Introduction of the GABP instead of the upstream NFκB site in the promoter of subtype B has previously been described [55]. An additional construct was made converting the unique GABP site in the subtype AE LTR into a second NFκB site. Plasmid pBlue3'LTR AE [22] was used as template in two independent PCR reactions under standard conditions. PCR primers 5' TA*G GGA CTT TCC *GCT GGG GAC TTT CC3' and 5'TGT CTC ATG AGC GGA TAC ATA3' were used in reaction A (italics indicate the NFκB-II site). Reaction B was performed with primers 5'GTC CCC TGC *GGA AAG TCC C*TA GTT AG3' and 5'TGG AAG GGC TAA TTC ACT CCC3'. Both PCR products, purified from gel, were used as templates in a third PCR under standard conditions with primers 5'TGT CTC ATG AGC GGA TAC ATA3' and 5'TGG AAG GGC TAA TTC ACT CCC3'. The 833 bp PCR product was digested with *Bse*A1 and *Hin*dIII, purified and ligated into pBlue3'LTR. The mutated subtype AE LTR was cloned from pBlue3'LTR into pLAI [57] using the *Xho*I and *Bgl*I restriction sites and verified by sequencing.

### Quantitative TaqMan assay

TaqMan assays were used to quantify the number of HIV-1 DNA copies in infected cultures. In brief, cells were resuspended in Tris-EDTA (10 mM pH 8.3) containing 0.5 units/μl proteinase K (Roche Applied Science), incubated for 1 hour at 56°C and 10 min at 95°C and directly used for PCR amplification. The number of input cells was determined using TaqMan^® ^reagents for quantification of β-actin DNA (AB, Applied Biosystems) according to the manufacturer's instruction. HIV-1 DNA was detected with a semi-nested real-time PCR assay with a pre-amplification step that is exclusive for completely reverse transcribed HIV-1 DNA. The pre-amplified product was subsequently quantified by real-time PCR as previously described [62].

## Competing interests

The authors declare that they have no competing interests.

## Authors' contributions

RMvdS was responsible for the majority of the experimental work, data analysis and drafted the manuscript. GP and MLvG have contributed to the experimental work. BB has contributed to the study design and writing the manuscript. REJ was responsible for the study design and writing the manuscript. All authors read and approved the final manuscript.

## Supplementary Material

Additional File 1 **Figure S1 HIV-1 activation from proviral latency over time**. SupT1 T cells were infected with the different subtypes. On day 2, 7 and 14 the cells were induced with TNFα (A), Vorinostat (B), mock treated or passaged and cultured for another week, followed by a repeat of the protocol. The cells were harvested 24 hours after treatment (day 3, 8 and 15, respectively) and analyzed by FACS for CA-p24 positivity. The fold activation from latency increases over time for all the subtypes except AE. Click here for file

Additional File 2 **Figure S2 Latency in the Jurkat T cell line**. Jurkat cells were infected with subtype B or AE in the format of the latency assay. **A: **Percentage of CA-p24 positive cells without inducer. **B: **The TNFα induced fold activation from latency. The results are presented as the average values of three independently produced virus stocks of which each stock is used for two independent infections. P values: *** = p < 0.001. Click here for file
