# Supplementary figures and images for: Latency profiles of full length HIV-1 molecular clone variants with a subtype specific promoter
Source: Retrovirology. 2011 Sep 16;8:73. doi: 10.1186/1742-4690-8-73 (PMC3182984; doi:10.1186/1742-4690-8-73)

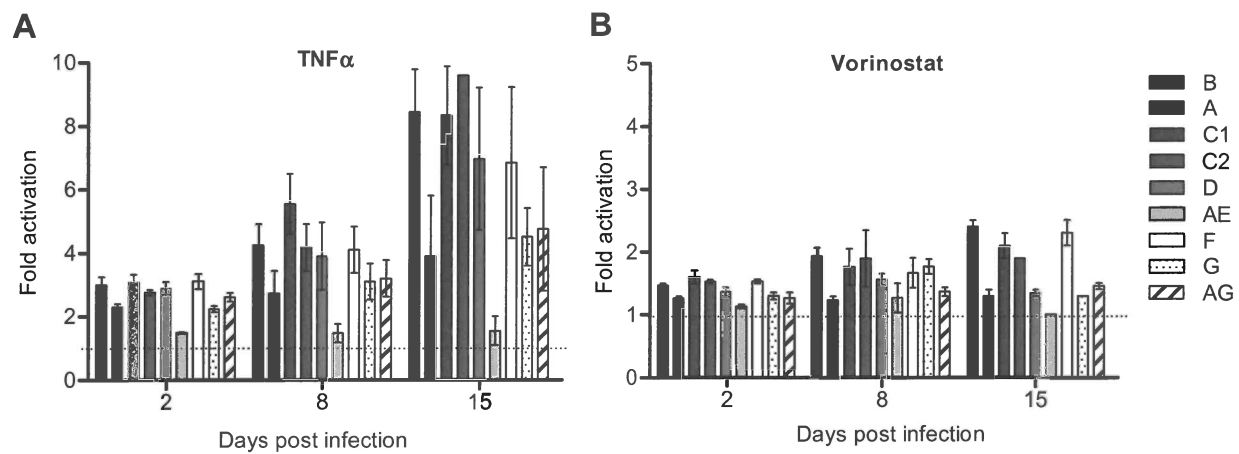

Supplement: Additional File 1 — Figure S1 HIV-1 activation from proviral latency over time. SupT1 T cells were infected with the different subtypes. On day 2, 7 and 14 the cells were induced with TNFα (A), Vorinostat (B), mock treated or passaged and cultured for another week, followed by a repeat of the protocol. The cells were harvested 24 hours after treatment (day 3, 8 and 15, respectively) and analyzed by FACS for CA-p24 positivity. The fold activation from latency increases over time for all the subtypes except AE. [file 1742-4690-8-73-S1.PDF]
