# Supplementary material for: Latency profiles of full length HIV-1 molecular clone variants with a subtype specific promoter
Source: Retrovirology. 2011 Sep 16;8:73. doi: 10.1186/1742-4690-8-73 (PMC3182984; doi:10.1186/1742-4690-8-73)
Supplement: Additional File 2 — Figure S2 Latency in the Jurkat T cell line. Jurkat cells were infected with subtype B or AE in the format of the latency assay. A: Percentage of CA-p24 positive cells without inducer. B: The TNFα induced fold activation from latency. The results are presented as the average values of three independently produced virus stocks of which each stock is used for two independent infections. P values: *** = p < 0.001. [file 1742-4690-8-73-S2.PDF]

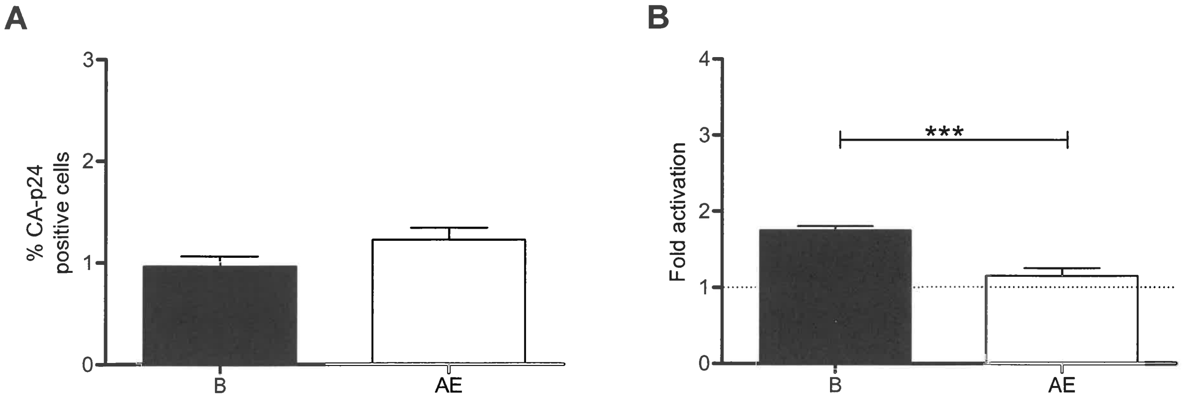

Supplementary Fig. 2 Latency in the Jurkat T cell line. Jurkat cells were infected with subtype B or AE in the format of the latency assay. **A:** Percentage of CA-p24 positive cells without inducer. **B:** The TNFα induced fold activation from latency. The results are presented as the average values of three independently produced virus stocks of which each stock is used for two independent infections. P values: *** = $p < 0.001$

Van der Sluis et al Suppl Fig 2
